# Beobachtungen; or, Observations on Original Malformations, &c.

**Published:** 1834-07-01

**Authors:** 


### Beobachtungen, &c.; or Observations on the Original Malformations and total Want of the Eyes in Men and in the Lower Animals. By *Von Seiler*.

[SECOND ARTICLE.]

In our last Number, we finished the third division of the work under review, and we now proceed with the fourth, and to the conclusion of our analysis.

### IV. Congenital Absence of both Eyes.

M. Botin has related in the History of the Royal Academy of Sciences, at Paris, 1723, an interesting case of an infant which lived for 41 days, and in which there was no trace of eyes. The lids had grown together, except at one point close to the inner canthus; upon dividing them, he found that the child had little or no power of moving them, and that no tears flowed out. The sockets contained nothing but a prolongation of the investing membrane of the lids, which was of a highly red colour and very sensitive to the touch. Almost all the cases on record of anophthalmus presented other deficiencies or malformations, besides those of the eyes, as, for example, fissures of the nose, lips, and palate—absence of the mouth, ears, &c.; in not a few, the number of fingers and toes was abnormal, sometimes exceeding the proper number, sometimes falling short of it. Those who wish to have a reference to most of the German cases will do well to consult Dr. Seiler's work. Some of the anophthalmous children lived for several months, others were still-born—in most, the eyelids and eyelashes were present, and indeed normal, with the exception of their being more firmly adherent to each other, and of being drawn inwards into the almost empty sockets.

It is much to be regretted, that accurate anatomical descriptions of the state of the brain and rest of the nervous system have not been given, by the numerous authors who have recorded cases of this monstrosity. Even those which have been published within the last ten years are equally imperfect in this respect. We shall attempt to gather together the histories of a few in which a dissection was made. Malacarne examined the body of an infant which lived for two months, and in which no trace of either eye was present. The eyelids were well formed, and could be freely moved during life at will; the sockets contained nothing but a red-coloured membrane, very like in appearance to the substance of the gums; the lachrymal glands, the puncta lachrymalia, and the lachrymal canals, as well the the Meibomian glands, were well formed. On dissection, it was found that the eyeballs with their muscles, the 2d, 3rd, 4th, and 6th pair of nerves, and optic thalami, were entirely awanting; there were no optic foramina in the sphenoid bone. The preparation is preserved in the Museum of Pavia. In two similar cases, related by Schmidt and Osiander, the optic nerves, thalami and foramina, were found wanting.

Schoen, in his Manual of Pathological Anatomy of the Eye, published at Hamburg, in 1828, describes the appearances which he found on dissecting an anophthalmous infant, which died in convulsions five days after birth. The eyebrows, lids, and lashes were well formed, but nothing except red lumps could be seen within the sockets. The 3d, 4th, 5th, and 6th cerebral nerves were found to be normal, but there were no optic nerves; otherwise, the brain itself did not exhibit any malformation. [It is to be regretted that this author does not particularize the state of the thalami and corpora quadrigemina.] The masses occupying the sockets of the eyes seemed to consist of cellular tissue and fat, and indistinct bundles of muscular fibres might be seen. The levatores palpebræ superioris were by far the most perfectly developed.

The anophthalmous child examined by Klintosh was also hydrocephalous. The ossification of the cranial bones was very imperfect; the forehead formed a large tumor, which projected anteriorly; the nose was awanting; the mouth misshapen, and there were no traces of most of the facial bones; the left eyeball looked like an hydatid, projecting between the two eyelids; the right one was quite awanting. On dissection, the brain did not exhibit any division into lateral hemispheres or lobes. The two lateral and the third ventricles together constituted a large sac, which held about nine ounces of clear serum. The body of the fornix was to be seen at the upper part of this sac, but no traces of its septum could be made out. The pineal gland and the corpora quadrigemina, were scarcely distinguishable; the corpora striata and thalami optici were rather more so, but still very imperfect. From the third ventricle, there proceeded a wide passage into a sac, formed by the dura mater; this projected through the substance of the sphenoid bone into the cavity of the mouth, and in which the pituitary gland was contained. The first six pairs of cerebral nerves were altogether awanting; the other pairs were present and apparently normal. Although the trunks of the fifth pair were wanting, many of their branches, dispersed on the parts exterior to the skull, were found as usual.*

The last case which we shall adduce was published by Daniel, of Halle, so far back as 1765. The fœtus was in the 7th month and hydrocephalic, the head being so disproportionally large, that it nearly equalled all the rest of the body. The spine, too, was bifid, and no doubt the vertebral canal was distended with fluid. The eyelids were found closed, and there were no traces of any eyes. The nose was exceedingly small and rudimentary; the left ear was awanting; so also were both the upper extremities; and the neck, chest and abdomen appeared externally as one continuous misshapen mass, there being no obvious distinction between these parts; some of the ribs could, however, be felt on the fore part and the spinal column posteriorly. On the upper half of this fleshy mass, which represented the neck, chest, and abdomen, was seen the attachment of the umbilical cord. The inferior extremities were moderately well formed; some of the toes were grown together. On dissection, the heart, lungs, trachea, diaphragm, liver, and the left kidney, were found to be awanting; only the right kidney, stomach, and intestines were present. The state of the spleen and supra-renal glands is not mentioned.

It is truly strange, that the recorder of the preceding case was so destitute

* This last-mentioned fact especially ought to be remembered, as it furnishes one of the most potent arguments against the hypothesis of the excentric development of animal bodies.

of scientific curiosity as not to examine the nervous system, for not a word is said in the report of the state, either of the brain or spinal cord. Two chalk drawings of this monstrous fœtus are given in Dr. Seiler's work.

That variety of anophthalmus which has been called a. cyclopicus, from there being but one median orbit under the frontal bone, is of more rare occurrence than the anophthalmus duplex. Prochaska has, however, left a description of an authentic case of the first of these malformations. The child was still-born at eight months; the eyelids of both sides were almost united to each other, and could scarcely be opened at all. Behind these was found only one orbit, but no eyeball existed within, and it was filled up with cellular tissue, fat, and a few muscular fibres. There was, moreover, no trace of any external nose, or of a mouth; the lower part of the face terminated in an obtuse point, on which was situated a small warty excrescence, underneath which there was an aperture, which led into the cavity of the mouth. The auditory and gustatory organs were also extremely misshapen, and some of them altogether deficient.

Clozoure has detailed, in the Revue Medicale for 1830, the particulars of a case, in which a single median or Cyclopic orbit, situated at the root of the nose, contained merely two small reddish warts. Over the upper eyelid, there was a prolongation of the skin, somewhat resembling an elephant's proboscis, and having a passage through it which led into the frontal sinus, and gave vent to a mucous discharge. The Royal Anatomical Museum at Berlin contains a specimen of a female monstrous fœtus, whose eye is indicated by an interpalpebral fissure, beneath the snout or proboscis. In some anophthalmous monsters, we find two orbits so closely applied to, or even, as it were, amalgamated with, each other, that we at once recognize in them the transition step to perfect cyclopia. In such cases, we usually find a mere skinny projection placed under the eyelids, and provided with no nostrils in the place of the nose. The particulars of a case of this description are detailed by our author: most of the cranial bones were abnormally formed; the æthmoid one was awanting, and where the cribriform lamina ought to have been, there was a cleft or gap, over which the dura mater was extended. Moreover, the nasal bones and the nasal processes of the superior maxillæ were deficient. The brain, in this case, was very small and soft, and exhibited indications of having been, at an earlier period, affected with hydrocephalus; there was no vestige of any convolutions, and the different protuberances, as the corpora striata, optic thalami, and so forth, were not at all distinguishable. The olfactory, optic, 3d, 4th, 6th, and 1st branch of the 5th cerebral nerves were awanting; the other pairs, including the other two divisions of the trigeminus, were present.

Another specimen of an anophthalmous Cyclopic monster is preserved in the Dresden museum. The eyeball, with all the appendages of muscles, bloodvessels, nerves, lids, lachrymatory apparatus, and also the nose, are awanting, and only at the place where the left interpalpebral fissure should be, there is a scar-like line. On the upper part of the head, there is a proboscideal protuberance, covered with hairs, and from this place, the skin is continued smooth down over the face, without any trace of eyelids or nose; the mouth is small and round. The proboscis has no external aperture, yet it is hollow, and the canal is continued downwards and backwards into the fauces, and upwards into the cranial cavity. The frontal bones, the small wings of the sphenoid, the æthmoid, and the inter-maxillary bones are awanting; the anterior edges of the parietal are nearly joined to the great wings of the sphenoid, leaving only between them a small interval, which is filled up with cellular substance, and covered over with the dura mater. It is worthy of notice, that in this, as well as in the preceding instance, the growth of the hair seems to have followed the development of the parietal bones. The only part of the encephalon that is quite perfect in its formation is the medulla oblongata. The cerebellum is indeed large, and exhibits the parallel windings on its surface; but its internal structure is exceedingly soft, and so watery that it resembles more a medullary cyst, than the ordinary structure. The first, second, third, fourth, first branch of the fifth, and the sixth cerebral nerves are awanting; the others are present.

At this stage of our inquiries it is important to remark, that, independently of any primitive malformation, the eyes of a fœtus may be so much destroyed by disease, that they are quite useless. Thus Roederer mentions a case of a hydrocephalic child, in which he found the eyeballs so squeezed, compressed, and altered from an accumulation of fat, and water within the orbits, that it was quite impossible to make out any of their component parts. Beer also alludes to some cases of congenital disease, in which the internal structures of the eye seemed to be quite confused and jumbled together, so that not one could be accurately distinguished. These morbid eyes were, however, not altogether insensible to the stimulus of light. But the limits of our paper prevent us from following out this subject. Hitherto we have alluded to the occurrence of anophthalmus only in the human fœtus; but examples of both kinds of it are not unfrequent in many of the lower animals also; as in the pups of the dog, cat, sheep, swine, &c. Tiedemann has recorded, in his Journal of Philosophy, an interesting dissection of an anopthalmous dog-pup. The diminutive and very short eyelid covered two exceedingly small orbits, which were occupied with cellular substance only. The inner surface of the lids was invested with a smooth covering, which was like the conjunctiva in appearance. On examining the brain, there were found in the place of the optic nerves, two extremely delicate fibres;—these proceeded from the thalami and corpora quadrigemina, and passing round the crura cerebri, terminated in front of the pituitary gland, without forming any commissure or union. The other ophthalmic nerves, viz. the third, fourth, and sixth pairs, were quite awanting. The brain, including the thalami optici and corpora quadrigemina were normally formed.

Sir A. Carlisle has recorded in the Philosophical Transactions for 1801 the dissection of an anopthalmous cyclopic lamb; and Meckel has related a similar case occurring in the swine. But it is unnecessary to multiply examples of an anomaly which is known to most anatomists. We shall therefore proceed to the more instructive task of enquiring whether we can account for the complete deficiency of such an important organ as the eye, upon the same principle as we have endeavoured to unfold and illustrate throughout this paper; we mean, the hypothesis of an arrest of or impediment to the ulterior development of the part, beyond its normal condition, at definite early periods of embryotic life. At the time when the dorsal laminæ form towards the cranial end, the small gaps or hollows for the cerebral vesicles, and before the anterior hollow, which is the widest, and is destined for the orbits, has been formed, we may suppose that from some cause or another the progress of the development of this latter hollow is arrested, so that while the posterior hollows, which are to receive the cerebral vesicles, are gradually but imperfectly formed, the optic ones remain in abeyance, and that at the same time the development of the nasal and oral cavities, (which may be regarded as formations which close the spinal laminæ anteriorly,) is impeded. Now very generally along with these defects, the anterior cerebral vesicles are either not at all, or are only partially formed;—thus it is that the anterior lobes of the brain, and, in connexion with these, the frontal bones, are found to be only rudimentary or are altogether wanting. It is curious to observe that the almost complete deficiency of the upper and fore part of the frontal bone is attempted to be compensated for by the unusually expanded size of the parietal bones, stretching from the occipital bone even to the rudiments of the superior maxilla, and of the malar bones; and laterally to the petrous portions of the temporal bones; while the place of the orbitar plates is supplied in some degree by a similar enlargement of the small wings of the sphenoid bone.

Not less interesting is the fact, that the extent of the hairy scalp seems always to correspond with the extent of the amplified parietal bones, even when they reach as far as the superior maxillæ and the beak-like rudiments of the nasal bones.

As the growth of the hair is limited in the normal condition of the parts to the integuments covering the cranial bones, and follows a line from the occipital protuberance round by the junction between the squamous and petrous portions of the temporal bones, and across the fore and upper part of the head, in the course of the coronal suture; so in the cases of monstrosity, which we have been speaking of, when the parietal bones appear to occupy the place of the frontal bone, and also of the squamous portion of the temporal bone, we find that the hairy scalp is equally increased in extent; and, in short, that the extent of the latter is uniformly in accordance with that of the former.

It may be supposed that, whenever an organ of sense is awanting, the cerebral nerves which belong to that organ will remain undeveloped; but this is not strictly the case;—true indeed there is invariably some malformation of them, either as regards their texture or their course; they may, for example, proceed a short way from the brain, and then suddenly stop short, the neurilema becoming blended with the adjacent dura mater; or the sheaths alone are found, and there is no medullary substance within them. But although the issuing nerves be imperfect, it is still possible that the portions of the brain whence they are given off may be quite normal, and yet the organs of sense to which the nerves belong may be altogether deficient. The researches of Tiedemann, as unfolded in his admirable work on the anatomy and development of the brain, have contributed most essentially to the elucidation of many of the most perplexing phenomena connected with cranial monstrosities.

That our readers may form some idea of their value, we shall briefly allude to the history of a monster figured in Dr. Seiler's plate, in which the whole of the face, (including the eyes, nose, &c.) between the os frontis and the lower jaw, were wanting; there was thus a large external opening leading right into the fauces. Now in this case, a person might have expected to have found that most of the cerebral nerves were wanting, as the organs, for which they are intended, were not present; and he may therefore be surprised to hear, that the only one which was altogether deficient was the olfactory; and the brain, with the exception of the greater part of the anterior lobes being wanting, was normal. Now Tiedemann tells us that such is very nearly the natural state of parts in a three months' embryo, every part of the brain being formed then, except the anterior lobes, and all the cerebral nerves, except the olfactory, being present. We have already adduced facts to shew that the absence of any organ does not necessarily suppose the entire absence of the nerves intended for its use. The converse of this state is also occasionally met with; the eyes, for example, are found, and yet there are no traces of retinæ or of optic nerves. Dr. Seiler mentions the case of a hydrocephalic child, which he examined; the sheaths of the optic nerves did not contain any medullary fibres, and they were so thin and delicate, that they could scarcely be traced on to the eyeballs; and yet these organs exhibited all their component structures, with the exception of their retinæ and corpora vitrea; and again, in a microphthalmous fœtus, no optic nerves could be discovered, and yet considerable advances in the development of the eye had taken place. In like manner, the olfactory organs are sometimes found to be tolerably well-formed, while the olfactory, and even also the trigemini nerves have been wanting.

There still remains another important theme of reflection suggested by the consideration of the preceding malformations. We have seen that when the eyeballs are awanting, many of the accessory appendages of these organs, such as the lachrymal glands, puncta, and canals, are also not unfrequently absent, just in the same manner as we find that, when there are no testicles, the scrotum, prostate, and so forth, are either not at all, or only imperfectly developed. But we are not to allow ourselves to conclude from such occasional occurrences that there must be therefore a law or invariable operation of the organizing power, by which one part is formed in consequence of another part, or by which the different organs are evolved from the centre to the periphery. This has been a favourite speculation of some late anatomists; but most assuredly it is contradicted by the best ascertained results of embryology. The position laid down by Rudolphi is much more correct;—"that every part of the centre and of the periphery is formed after its own definite and distinguishing type, according to the period of its development, and either primarily in its own locality, or to be subsequently affected by time or by situation, provided all along that no impediment or arrest ever take place." This holds true, in an especial manner, with the development of the nervous system. Klinkosch found, in a case where one of the trunks of the trigeminus was entirely deficient, its peripheral ramifications in their proper localities; and Dr. Seiler adduces his own observations in proof of the accuracy of the statement. He examined a fœtus, in which, as in the example related by Rudolphi, all the nerves were present, although the brain and the spinal marrow were altogether awanting. In consequence of a few of such cases, M. Serres, in his work on the Comparative Anatomy of the Brain, published at Paris in 1824, announced the doctrine that the development of the nerves commences at the periphery, and proceeds to a central point, at which all meet; but the whole history of embryology, from the first month until the perfect formation of all its organs, the earlier formation of the trunk, the subsequent formation of the head, and of the extremities, (these two shooting from the centre peripherally) and lastly every instance of regeneration stand opposed to M. Serres' hypothesis. The tendency of the developing power is always from the centre towards the periphery; parts which are intermediate may indeed be arrested in their formation, while the parts on either side may advance in their normal progress; it is in this way that we explain the rationale of those cases in which the branches of a nerve are developed, while the trunk of the nerve is awanting, or in which the rudiments of the forearm and hand are found attached to the shoulder-blade, while the upper arm is altogether absent. Other examples illustrative of the same doctrine may be adduced from the occasional occurrence of a trunkless head superimposed upon another head; or of a bicephalous monster, or of the complete absence of the cervical vertebræ, the head being then joined on to the dorsal vertebræ, &c. and still more striking proofs are those strange anomalies of organisation, in which one quarter, or even one entire half of the body is deficient, as for example, the head, chest, and part of the belly, while the other quarter, or other half is regularly formed. Surely such monstrosities are quite irreconcileable with Serres doctrine of concentric or converging development.

### Part II.—Of the Original Malformations of Special or Individual Parts of the Eye.

*Orbits.* In the preceding part of this paper we have spoken of most of the congenital defects of these cavities. Whenever the head is much enlarged by hydrocephalus at birth, it has been found that the orbitar plates of the frontal bone have generally their oblique, or nearly horizontal position altered to a more perpendicular plane, and that thus the size of the orbits is necessarily reduced. If again the development of the olfactory organs be imperfect, or irregular, the situation and shape of the orbits may be affected in consequence; thus they may be placed too close to each other, or they may be even melted into one, constituting the monophthalmus cyclopicus. A variety of other malformations is occasionally met with; but the consideration of these need not detain us longer. It is important to observe that in many cases of microcephalus the orbits are not only proportionally too small, but they even want one or more of their component bones.

*Eyelids.* The shortness of the lids, when they are insufficient to cover the globes entirely (lagophthalmus), may very often be referred to an interruption of the development of these parts. Sometimes the lids are altogether awanting, although the eyeballs be perfect. Morgagni alludes to his having seen a specimen of this deformity in a man whose corresponding eye was quite destroyed; but he does not mention whether this was congenital or not. The deficiency of the lids in anophthalmous monsters is by no means infrequent. The adhesion of the eyelids to each other (anchyloblepharon) or to the conjunctiva of the ball, (symblepharon) is probably oftener the effect of an intra-uterine inflammation, than of any original malformation. That inflammations of different organs of the fœtus do occasionally take place, we can have no rational doubt, when we consider the numerous instances on record, authenticated by good witnesses. Chaussier found in a seven-month fœtus most distinct traces of enteritis and diaphragmitis. Vernan mentions having seen in the body of a new-born child a quantity of sero-purulent effusion within the pleuræ, which were at the same time coated with lymph. The thymus has been found inflamed, and Otto in his Manual of Pathological Anatomy says that he has seen several well-formed and very obvious ulcerations on the lips and gums of a new-born infant. The conjunctiva oculi, in cases of congenital deficiency of the lids, has been noticed by several observers to have become thick and red; and this change is sometimes so striking, that the membrane has assumed the appearance of the membrana nictitans of birds.

It cannot then be questioned that the eyes, or some parts of them may become involved in disease, while the fœtus is in utero; and it will sometimes require a practised observer to determine whether the abnormal appearances of the organ at birth are to be attributed to disease, or to a primitive malformation. Ectropium and entropium occasionally occur as congenital maladies; and also blepharoptosis, in which the upper eyelid from its unusual length hangs down over the lower one. In cyclopic monsters, there are sometimes rudiments of the four lids placed round the single eye; at other times there are none at all.

*Lachrymal Organs.* In our account of anophthalamus, it may have been observed, that there is no steady rule with regard to the presence or absence of these organs in cases of this congenital defect. In Klinkosch's case, they were awanting; but not so in that related by Rudolphi; and in an example which occurred to Weidele, the lachrymal gland was found lying in the place of the absent eyeball. Again, the lachrymatory apparatus may be either entirely or partially deficient, although the eye has been formed.

Cyclopic monsters very often present rudiments of the double formation of many of the ocular appendages; and among these, of the lachrymal gland.

*Muscles of the Eyeball.* It would be very interesting if we were better informed respecting the congenital malformations of these muscles, as probably considerable light would thereby be thrown on some of the causes of squinting (strabismus and luscitas). Wrisberg found in a man who had squinted greatly, the cornea to be higher up than natural, one of the recti muscles wanting, and the others to be grown together. Otto mentions the occasional shortening or even entire absence of some of the recti muscles in such cases. Dr. Seiler states that he dissected a case in which the two oblique muscles were wanting, although the eyeball and other parts were normal. Sometimes, but very rarely, more than the usual number of ocular muscles have been found. Kulmus and Meckel have met with two upper oblique muscles, which had separate origins and separate pullies, and Caldani has described a muscle which arose from the lower edge of the orbit and expanded itself on the lower lid.

The duplicity of the muscles is very common in cyclopia.

*Nerves.* Our notice of this head need be short, in consequence of our having repeatedly throughout this paper alluded to the condition of the ocular nerves in malformations of the eyeball. We have seen that some or all of them may be either entirely wanting or that they may be reduced to mere membranous cords, having no medullary substance within. An interesting irregularity is occasionally met with; viz. where the optic nerves run apart from each other throughout their whole course, from the thalami to the eyeballs: now, although such an arrangement has not hitherto been shewn by any anatomist to be normal at any stage of embryotic life, it points to a defect of the organising principle in joining the two lateral halves of the body. Numerous instances are on record in which this malformation was found, and yet the brain and adjacent parts were otherwise quite regular. It is by no means infrequently observed in microcephalous monsters. In a cyclopic dog-pup, Majendie could find no trace whatever of any optic nerve; the brain was exceedingly small, without any convolutions, and lay like a simple tubercle in the back part of the cranium. The first five pairs of cerebral nerves were quite wanting. A very similar case is related in Haller's lesser works. The converse of such occurrences is also sometimes met with; the eyeball being absent, while the optic nerve has been normally formed. This nerve, like most other parts of the eye, is usually, but not always, double in cylopic monsters; and in such cases an irregularity of the entrance of the nerve or nerves is not uncommon.

*Sclerotica and Cornea.* The former of these membranes is, in the young fœtus, and even in children at the birth, very thin, and of a blueish-white colour; and if from any cause the perfect development of the eyeball has been impeded, this state of the sclerotic remains through life. In the third and fourth months of fœtal existence, the sclerotic is of an oval or lengthened shape, in consequence of its anterior part projecting very much forwards at these periods. This prominence [protuberantia scleroticæ fœtalis] becomes gradually less and less, so that by the ninth month it is not very apparent. In microphthalmous fœtuses it is generally most distinct. In them the cornea often remains cloudy, and not easily to be distinguished from the sclerotic; it has a lengthened shape, and appears to be surrounded with a purplish ring. Many of the congenital affections of this membrane which have been attributed to fœtal disease, are in fact only signs of a retarded development. Thus authors have often spoken of the cornea being found altogether awanting, the sclerotic being then continued over the front of the eye; probably this anomaly is always explicable on the above principle. In such cases it may almost always be observed that the supposed prolongation of the sclerotic is rather more thin and transparent than the rest of the membrane; and perhaps the most anterior or central point may be even moderately clear and permeable to the light. The example narrated by Kieser in Himly and Schmidt's Ophthalmological Dictionary seems to have been of this description. Dr. Farrar relates the cases of new-born triplets, in each of whom the cornea was quite opaque. In two the opacity had in a great measure disappeared within twelve months after birth; while in the third, no change had taken place at the date of the history, the child being at that time three years old. We must not however suppose that every case of congenital opacity of the cornea is referable to the cause now mentioned; for doubtless some are the effects of a previous inflammation in the part. Wardrop refers to several examples of congenital tumors growing on the cornea, and Baron Graefe has met with cases of telangiectasis, or nævus of this membrane. A much more common malformation is the extreme or morbid prominence of its convexity, constituting the affection which has received so many appellations; such as "staphyloma corneæ pellucidum, or hyperkeratosis," by Himly; "ochlodes," by Taylor; "conical-formed cornea," by Wardrop; "sugar-loaf cornea," by other English surgeons; "cornée conique," "staphylome transparent de la cornée," by most French authors; and "kerato-conus," by Ammon. The most projecting point of the convexity is seldom at the centre of the cornea; but generally somewhat to the side; and often it has a peculiar glancing lustre, like a piece of polished glass; or it may have even an opalescent play of colours; or on the other hand it may be dull and smoky. This affection ought to be always distinguished from the other and more common variety of staphyloma [s. corneæ opacum]; also from hernia of the cornea [keratocele] and from dropsy of the eyeball. If the degree of the protrusion be not very great, the child may retain its vision, but must be exceedingly short-sighted; and unfortunately if it be considerable, the patients are almost always completely blind. To Professor Jager of Wurzburg, we are indebted for the best anatomical description of this malformation; it appears from his researches that the cornea is often unequally thick at different parts, being much thinner than usual in one place, and much thicker in another. Some of the other structures of the eye also are occasionally involved; thus the pupil may be closed, or the lens may be cataractous; or the retina amaurotic. Ammon and Jager have remarked that this congenital affection of the cornea is very often associated with some peculiarity in the formation or shape of the cranium; for in some it was unusually small and conical; in others, the frontal bone was much flattened and depressed, while the parietal and occipital bones projected more than ordinarily. Now in connection with these facts it is most interesting to find that those able observers, Ammon and Gescheidt, have discovered hyperkeratosis in a two-month embryo; and the question therefore arises in our minds, whether the affection is to be viewed as the result of a retarded or interrupted development, or of a fœtal disease. Wimmer inclines to the former opinion, and supports it by referring to the condition of the eye, not only in those animals which are lower in the scale, but also in the young of all mammals, including man; the cornea being uniformly more conical during its fœtal development than it is afterwards. He says, "many physiologists have ascertained that the human embryo, from the period of its earliest to that of its perfect formation, gradually passes through several stages of organization, each succeeding one being of a higher grade than that which went before it.

Now in many animals, as in the genera astacus and lucanus, among insects; in most birds, especially in those which are carnivorous, and in some of the mammiferous class, as the mole and porcupine, a very great convexity of the cornea is the normal or healthy state."

But this merely analogical reasoning cannot be admitted as sufficient to resolve our doubts; and until it has been proved by competent authorities that the natural condition of the embryotic cornea is that of extreme prominence, we must hesitate before we arrive at any conclusion. Hitherto embryologists have not succeeded in determining this point: and if the weight of their testimony leans to any side, most assuredly it is unfavourable to Wimmer's view of the question. The observation therefore of Ammon and

Gescheidt, of the occurrence of this anomaly in a two-month embryo, must, in the present state of our knowledge, be considered rather as an example of a diseased, than of a merely interrupted condition of the cornea; but whether this membrane is primarily affected, or only consecutively, from an excess of the aqueous humour, is still very uncertain.

Although we have thus, in obedience to the strict laws of deductive reasoning, refused our assent to the doctrine maintained by Wimmer and others, in reference to the origin of hyperkeratosis, we must avail ourselves of this opportunity of urging the great importance of numerous anatomical examinations of this, and, indeed, of all other congenital ophthalmic affections; the development of the eyeball and of the brain stand in so close and so allied a relation to each other (according to Huschke, the former being only an expansion of the canal of the optic nerve, which proceeds from the cerebral bladder), that the irregularities of the one may very probably be supposed to be similar to, and also to influence, those of the other organ. As malformations of the cranial bones are more common in hydrocephalic children, so in hyperkeratosis, the structure of the other parts of the eye is more frequently abnormal. If the development of the cerebral mass, from its primitive watery condition, be unusually tedious and imperfect, we can readily understand the reason why the medullary matter of the nerves which issue from it is, in such cases, thin and pulpy, and why the retina, for example, should resemble a serous membrane more than a nervous expansion. We have strong reasons for believing that a great many of the cases of congenital amaurosis derive their origin from this cause. The partial thickening of the cornea in hyperkeratosis is probably owing, in the chief degree, to the pressure of the contained aqueous humour, just in the same manner as the cysts of many watery swellings, and even the bones of the cranium in hydrocephalic children, who have survived for a considerable time, are condensed. Loder has preserved in his museum the skull of a hydrocephalic child, two years old, in which the parietal bones are three quarters of an inch thick, and in the case of a patient who lived 42 years, Schneider found one of the parietal bones an inch and a quarter in thickness. A similar remark often holds true, with respect to the membranes of the eye, in other affections besides hyperkeratosis; for in dropsy of the eyeball, the sclerotic is very often found thicker than usual. In cyclopic eyes, the cornea is generally double, or at least exhibits traces of being so.

### The Choroid Coat—Corpus Ciliare, and the Pigmentum Nigrum.

The choroid coat, as well as the retina, has been found cleft by Ammon and Gescheidt in the eye of an adult person; this imperfection (coloboma choroidea) is now known to be the normal state of the membrane during one period of embryotic life.

When the pigmentum nigrum is only partially deficient, the eye of the individual is rather more than ordinarily susceptible to the stimulus of light; and this state may be considered as the transition-stage between health and albinism. Our limits prevent us from following Dr. Seiler, in his interesting account of peculiarities of body which characterize the latter curious affection, and we must limit our remarks to the appearances of the eyeball only. The conjunctiva is usually very pale, or it may have a somewhat red aspect, from the bloodvessels which permeate its structure; the sclerotic is thinner, and has a purplish hue; the cornea is generally more convex, the choroid is without its pigmentum, and the iris without its uvea. The iris is usually of a blueish-red colour, the blue prevailing towards the inner, and the red towards the outer circumference; and the surface of this curtain often exhibits a beautifully striated structure, the striæ diverging from the pupil to the outer circle, and being interlaced and connected together by means of transverse striæ or fibres, especially towards the pupillar margin. If the eye be examined when a bright light is permitted to fall upon it, the pupillar part of the iris appears of a pale blue colour, and the outer or ciliary part exhibits a red tinge, which glimmers between the white striæ. The red colour is scarcely perceptible when the pupil is dilated, as in the shade. In most albinos, the eyeballs are perpetually moving to and fro, from one canthus to the other, and the iris is often in a continual state of oscillation. Short-sightedness is very common among albinos. Albinism, as is well known, is sometimes only partial, large patches of the surface of the body here and there being quite white, while the rest retains its natural colour; the eye may be similarly affected. Rudolphi saw a dog, in which one half of the iris was white, and the other half quite dark; but in two or three histories of piebald human albinos which are on record, it is stated that the eyes were black throughout, and, therefore, that they did not exhibit the irregularity alluded to. The proximate cause of albinism, (or, as it has been called by different authors, leucœthiopia, leucopathia, leucosis, or the white malady) is indubitably a deficiency of the colouring matter of the skin and of the eyes; but if we enquire what has probably given rise to this deficiency we shall find ourselves at once perplexed between two theories—the one assuming that it is really, and in truth, a morbid state, or peculiar cachexy, somewhat analogous to the disease known by the names of lepra alba or alphos, and having for its essential character the partial or complete absence of the ordinary colouring secretion, which ought to be deposited between the layers of the skin and on the surface of the choroid coat; Blumenbach, Winterbottom, Sprengel, and others have taken this view, while, on the contrary, Hallé, Jefferson, Beclard, Mansfeld, and Meckel (although the last-named author hesitates to decide), regard albinism as the effect of an arrest of the normal development. If, indeed, we include under the term "cachexy" every irregularity or abnormal condition of the system proceeding from a deviation from the usual combinations of the solid or fluid parts, there can be little doubt that leucosis must be reckoned as such, especially since we know that even adult animals may be so affected by certain causes, inducing a depraved state of the system, that the colour of the skin and hair, becomes speedily changed. Geoffroy St. Hilaire has satisfied himself, by repeated observations, that many animals, and particularly those of the genus simia, when long confined, and when their food is not quite wholesome, gradually lose their wonted colour, and fall into a state of imperfect leucosis. He found, also, that young gold fishes, if kept for some weeks in spring water, became nearly quite white. Every one has heard of the extraordinary effects of a sudden paroxysm of grief and terror in blanching the hair; and the almost constant occurrence of "silver locks" in old age, even among the dark races of mankind, seems to point to the operation of certain changes being effected within the animal system.

But certainly it is most erroneous to draw any analogy between leucosis and any exanthematous disease, such as lepra; for the skin of an albino, although of a pearly whiteness, does not exhibit any visible morbid change, save that of its colour; there are no scales, no excrescences, and there is no oozing of any discharge. In some parts, indeed, as in the Island of Sumatra, the albinos have been found affected with the leprosy—but this was a casual, not an uniform occurrence. If, therefore, leucosis be a disease, it is a disease sui generis, and one, too, which is peculiar to fœtal life; for no instance of complete albinism, supervening after birth, in an individual previously unblemished, has been ever recorded.

What adds exceedingly to the difficulty of ascertaining positively the remote cause of this anomaly, especially in the human subject, is the rarity of opportunities which ever fall to the lot of any anatomists, of examining very young embryos; and until our knowledge of the various steps or grades of development through which the intra-uterine being passes, before it arrives at its perfect formation, be much more exact than it is at present, there must be of a necessity considerable uncertainty on most embryological speculations. Dr. Seiler has been led to give the preference to the latter of the two theories above mentioned, by the following considerations.—1, Although the pigment of the eye is deposited at a very early period of embryotic life, it is not uniformly or equally so on the whole surface of the choroid and of its appendages; by far the greater portion being accumulated, at first, on its anterior part and on the ciliary processes, while the posterior part is almost quite free from it, and retains, therefore. a red colour; such an appearance is occasionally observable in the eyes of hydrocephalic children. There is, therefore, reason to suppose, that the pigment is altogether awanting in the very early stage of fœtal development. 2, In the eyes of piebald horses, a very evident proof of an arrested formation is exhibited; for, in these, the pigment is limited to the corpus ciliare. 3, The skin of the embryo, in the first month, is destitute of any traces of pigment. 4, Most of the infusoria (those animals which are at the lowest step of the zoological series) are colourless, or have only a greenish hue. 5, The soft white hair (lanugo) of the albino, which remains with him during life, is analogous to the capillary system of a five-months' fœtus. 6, The debility of body and of mind, which so very frequently characterizes albinos, is indicative of imperfect development of the natural powers. 7, It has often been remarked, that albino children have been in many instances the offspring of feeble and exceedingly delicate parents, whose formative energies we may, therefore, reasonably believe to be below the healthy standard. Be this as it may, no one can doubt the surprising effects which vehemently-depressing agencies will produce in altering, or even in altogether abolishing, the former colour of the body.

The limits of this paper prevent us from following our industrious author, in his description of the congenital malformations of the iris, uvea, &c. and leave us room only to allude to the occasional persistance of the membrana pupillaris, for several months, or even years, after birth. Probably, few of our readers are aware that it is the prevalent opinion among our professional brethren in Germany, that the operation of restoring the blind boy to sight, which has given so much eclât to the name of our countryman, Cheselden, was, in fact, that of forming a pupil through the persistent pupillar membrane, and not the operation of couching, or depressing a congenital cataract. That, however, the lens has in numerous instances been found to be cataractous at birth, no one will deny, when he regards the names of Beer, Saunders and Ammon as vouchers for the fact. So long ago as the year 1810, Professor Walther, in his admirable treatises on medical subjects, threw out the idea that the lens was actually opaque during the early stage of embryotic existence, and that it became transparent only at a subsequent period; the inference from this doctrine is very obvious, viz. that in the event of it being proved to be correct, congenital cataract must be viewed rather as the effect of retarded development, than of positive disease. The later observations of Baerens, Ammon, and of Dr. Seiler, are, however, quite opposed to Walther's hypothesis, and do not at all warrant the supposition, that the lens is ever normally clouded. The researches of the second-named philosopher have induced him to trace the occurrence of congenital cataract to one of the following causes:—1, A morbid affection of the arteria centralis retinæ, or of its contents. 2, A thickening, the result of intra-uterine inflammation of the anterior, or of the posterior wall of the capsule of the lens. 3, A primitive disease, or dyscrasis, of the substance of the lens. Such a state may be connected with an hereditary taint, as that of scrofula.

That cataract may be transmitted from one generation to another will not be doubted, when the following case, authenticated by the testimony of Beer, is read.

The father had cataract—so also had his son. This son was twice married, and had families by each wife; and every child (the number amounting to 8 or 10), with the exception of one, became cataractous before their twelfth year.

Congenital cataract is sometimes only partial, or affects but one point of the lens, while the rest of it is healthily transparent. This opaque spot is usually circular, and is seldom obliterated in after life.

Two lenses have been sometimes discovered in a cyclopic eye; but there is no well-authenticated case on record, of the entire absence of all traces of the lens at birth.

Our abstract of Dr. Seiler's work must here finish, and we trust that, ere long, we shall find him once more in the list of authors. No one, who has perused his present performance as it deserves, will hesitate to acknowledge that he has derived much interesting and valuable information from it. It is an admirable compendium of all that is at present known, respecting the very curious and instructive subject of which it treats.

